# PPARs and Myocardial Infarction

**DOI:** 10.3390/ijms21249436

**Published:** 2020-12-11

**Authors:** Kay-Dietrich Wagner, Nicole Wagner

**Affiliations:** Université Côte d’Azur, CNRS, INSERM, iBV, 06107 Nice, France

**Keywords:** peroxisome proliferator-activated receptor, myocardial infarction, cardiovascular disease, angiogenesis, endothelial cells, cardiomyocytes

## Abstract

Peroxisome proliferator-activated receptors (PPARs) belong to the nuclear hormone receptor family. They are ligand-activated transcription factors and exist in three different isoforms, PPARα (NR1C1), PPARβ/δ (NR1C2), and PPARγ (NR1C3). PPARs regulate a variety of functions, including glucose and lipid homeostasis, inflammation, and development. They exhibit tissue and cell type-specific expression patterns and functions. Besides the established notion of the therapeutic potential of PPAR agonists for the treatment of glucose and lipid disorders, more recent data propose specific PPAR ligands as potential therapies for cardiovascular diseases. In this review, we focus on the knowledge of PPAR function in myocardial infarction, a severe pathological condition for which therapeutic use of PPAR modulation has been suggested.

## 1. Introduction

Acute myocardial infarction is one of the leading causes of death, the prevalence of the disease approaches three million people worldwide. The etiology of acute myocardial infarction is decreased coronary blood flow. The available oxygen supply cannot meet oxygen demand, resulting in cardiac ischemia. Risk factors include obesity, dyslipidemia, and diabetes mellitus. Activators of PPARs are used to treat a variety of metabolic disorders, like diabetes and hyperlipidemias, via individual or combined activation of PPAR isoforms. PPARs are ligand-activated transcription factors that belong to the nuclear receptor superfamily. They exist in three different isoforms: PPARα, PPARβ/δ, and PPARγ, and are activated by fatty acids and their derivates, linking them directly to metabolism. PPARα is mostly expressed in the liver, heart, brown adipose tissue, kidney, and intestine. PPARβ/δ is ubiquitously expressed with some species differences, and PPARγ is detectable in adipose tissue, the heart, gut, and immune cells. Synthetic specific agonists are available for all PPARs. PPARs heterodimerize with retinoid X receptors (RXR) and bind to cis-acting DNA elements, PPAR response elements (PPREs), which results in increased gene transcription [1]. Though therapies with activators of PPARs have mostly favorable effects on the risk factors for cardiovascular disease, also adverse effects on the cardiovascular system occur, which mitigates their advantageous effects, thus limiting their widespread use in patients with cardiovascular risk. Our review summarizes basic and clinical research findings associating PPARs with beneficial or detrimental effects in the setting of myocardial infarction.

## 2. PPARs and Myocardial Ischemia/Infarction

### 2.1. PPARα

In the late 1980s, the Helsinki heart study suggested the PPARα agonist gemfibrozil for the prevention of coronary artery disease [2]. At this time, it was not known that gemfibrozil actually was a PPARα agonist, as PPARα had been identified as the first PPAR in 1990 [3]. In the Helsinki heart study, the effect of modifying plasma low-density lipoprotein (LDL), and high-density lipoprotein (HDL) cholesterol on the primary prevention of coronary heart disease in middle-aged men with hypercholesterinemia was investigated over a five year trial period. A 34% reduction in the incidence of coronary artery disease had been observed [2]. In 1998, the bezafibrate infarction prevention (BIP) study was initiated. Bezafibrate is a lipid-lowering fibric acid derivate and a pan (α, β/δ, γ)-PPAR agonist. The aim of this trial was to investigate if bezafibrate would reduce the risk of myocardial infarction in coronary artery disease patients. An eight-year follow-up demonstrated a 17% reduction of major cardiac events [4]. 1999 was the start of the ACCORD (action to control cardiovascular risk in diabetes) trial, which aimed at elucidating whether combination therapy with a statin (simvastatin) plus fenofibrate, as compared with statin monotherapy, would reduce the risk of cardiovascular disease in patients with type 2 diabetes mellitus. Combination of the PPARα agonist fenofibrate and simvastatin did not reduce the rate of fatal cardiovascular events, nonfatal myocardial infarction, or nonfatal stroke, as compared with simvastatin alone [5]. Later, first experimental studies investigating the effects of PPARα modulation on the outcome of myocardial infarction emerged. In 2002, the group of C. Thiemermann was the first to examine the effects of PPARα agonists (clofibrate and WY14643) after experimentally induced myocardial infarction. They also investigated the effects of PPARγ agonists (thiazolidinediones and cyclopentanone prostaglandins) on myocardial infarct size [6]. The detailed information about the substances or experimental interventions used as well as the outcome on myocardial infarction for all PPARs, are summarized in Table 1.

The authors administered the different agonists 30 min before coronary artery occlusion (for 25 min) and reperfusion (2 h) in rats. Afterward, they determined the respective infarct sizes. Regarding the PPARα agonists, clofibrate reduced infarct sizes to about 30%, and WY14643 to ≈ 44% of that observed in control DMSO treated rats. In the group of thiazolidinediones, rosiglitazone, and ciglitazone caused the most significant reduction of infarct sizes (≈45%), whereas pioglitazone reduced the infarcted area to about 25%. However, the highest reduction of infarct sizes was observed when the animals were treated with PPARγ agonists cyclopentanone prostaglandins: 85% reduction with 15D-PGJ_2_, and ≈47% with PGA_1_. The authors proposed inhibition of the transcription factor nuclear factor kappa-light-chain-enhancer of activated B cells (NF-κB) as one mechanism of cardioprotective actions following pretreatment with PPARα and/or γ agonists, as they observed an increase of NF-κB pro-inflammatory target genes such as monocyte chemoattractant protein-1 (MCP-1) and inducible nitric oxide synthase (iNOS) after myocardial infarction, whose expression was attenuated in case of pretreatment with 15D-PGJ_2_. Furthermore, 15D-PGJ_2_ upregulated the expression of cardioprotective hemeoxygenase-1 (HO-1). However, although 15D-PGJ_2_ is a potent agonist of PPARγ, not all the effects observed seemed to be mediated via PPARγ. In vitro studies with rat cardiomyocytes evidenced that only 15D-PGJ_2_, but not the PPARγ agonist rosiglitazone was able to increase HO-1 expression. The authors suggested that the beneficial effect of 15D-PGJ_2_ could also be due to the simultaneous activation of PPARα [6]. Similar beneficial effects were also observed by Tian-Li Hue and colleagues. Using a mouse model system for ischemia (30 min) and reperfusion (24 hrs), they evaluated the effects of the PPARα agonist GW7647. GW7647 substantially reduced infarct sizes and improved myocardial contractile dysfunction. These effects were abolished in PPARα knockout mice, confirming the selectivity of the chosen agonist for PPARα. After ischemia/reperfusion, GW7647 attenuated the decreased myocardial fatty acid oxidation (FAO) enzyme activity, the release of pro-inflammatory cytokines, neutrophil accumulation, and inhibited NF-κB activation. These results further suggested the metabolic and anti-inflammatory properties of PPARα in cardioprotection [7]. Completely contrasting results were observed in a study by Sambandan and colleagues. Using transgenic mice with cardiac-specific overexpression of PPARα, wildtype, and PPARα knockout mice, the authors demonstrated using ischemia (18 min) and reperfusion (40 min) that PPARα knockout mice had significantly lower FAO, higher glucose oxidation rates, and better recovery of cardiac power (calculated as the product of developed pressure and cardiac output) than animals from the two other groups. In contrast, mice with cardiac overexpression of PPARα showed highest FAO, lowest glucose oxidation rates as well as worst recovery of cardiac power, indicating a detrimental effect of chronic PPARα activation on cardiac recovery after ischemia [8]. These findings were later confirmed by Duerr and colleagues, who demonstrated that cardiomyocyte-specific overexpression provokes irreversible damage in murine hearts subjected to repeated ischemia/reperfusion procedures. They observed higher glycogen deposition, increased apoptosis, disturbed antioxidative capacity, and maladaptation of contractile elements as major elements of the deteriorated ventricular function during brief, repetitive ischemia/reperfusion episodes in animals with cardiomyocyte-specific overexpression of PPARα [12]. In diabetic patients, several prospective, randomized, and double-blind clinical trials for the dual PPAR (α and γ) agonist muraglitazar in comparison with the PPARγ agonist pioglitazone and placebo were associated with a higher incidence of major cardiovascular events as myocardial infarction or stroke [37]. Later trials with Aleglitazar, also a dual PPARα and γ agonist, had to be stopped due to excess adverse events, making this class of therapeutics unlikely to be successful in the treatment of cardiovascular disease in diabetic patients [38]. The Fenofibrate Intervention and Event Lowering in Diabetes (FIELD) study, implying 9795 patients with type 2 diabetes mellitus, showed that fenofibrate did not significantly reduce the risk of the primary outcome of coronary events but reduced total cardiovascular events, mainly due to fewer non-fatal myocardial infarctions and re-vascularizations [39]. One experimental study elucidated the effects of chronic PPARα activation using fenofibrate in heart failure after induced myocardial infarction in rats. No changes in left-ventricular dilatation or dysfunction despite the enhanced cardiac FAO and left ventricular hypertrophy upon PPARα modulation were observed [9]. Similar findings were reported in fenofibrate treated pigs, which were submitted to ischemia/reperfusion: Myocardial infarct sizes, contractile function, lipid accumulation, substrate uptake, and expression of carnitine palmitoyltransferase 1 (CPT1), important in long-chain fatty acid oxidation, were unaffected by PPARα activation [10]. Two comparative studies linked single-nucleotide polymorphisms (SNPs) concerning PPARα in humans to a higher risk of myocardial infarction [40,41]. The PPARα agonist WY 14643 had cardioprotective effects in Goto Kakizaki rats (an animal model of diabetes mellitus type 2) upon ischemia/reperfusion manipulations. The authors observed reduced infarct sizes and increased serine/threonine-protein kinase (AKT) and endothelial nitric oxide synthase (eNOS) signaling in the ischemic myocardium of Goto Kakizaki rats treated with WY 14643 [11]. Similar findings were observed in non-diabetic rats submitted to myocardial infarction and treated with the PPARα agonist clofibrate. The authors focused in their analysis on inflammation related molecules and demonstrated reduced expression of interleukin (IL)-6, tumor necrosis factor (TNF)-α, intercellular adhesion molecule (ICAM)-1, vascular cell adhesion molecule (VCAM)-1, matrix metallopeptidase (MMP) -2 and -9, NF-κB, and inducible nitric oxide synthase (iNOS) upon clofibrate treatment. Echocardiographic examinations showed that clofibrate reduced left ventricular dilatation, however, other important functional parameters as left ventricular ejection fractions (LVEF) were not improved [13]. Cardioprotective functions of PPARα were also proposed in a recent publication where the potential therapeutic effects of ginsenoide Rb-3 (GRb-3) on the outcome after myocardial infarction in mice were examined. Animals were treated either with GRb-3 or fenofibrate. Both substances similarly increased ejection fractions (EF) and fractional shortening (FS), and inhibited apoptosis. The PPARα inhibitor GW6471 attenuated the effects of GRb-3 in species different rat cardiac myoblasts in vitro, suggesting that cardioprotective consequences of GRb-3 therapy might be mediated through PPARα activation [14].

The initial enthusiasm regarding the potential therapeutic benefits of PPARα agonists as activators of cardiac FAO and inhibitors of glucose utilization in the prevention and cure of myocardial infarction has not only been dampened by negative or not clearly beneficial outcomes in large clinical trials but also extremely contrasting results of experimental studies. The role of PPARα in myocardial infarction remains unclear as both beneficial and detrimental effects of PPARα activation have been reported. This might be due to ligand-dependent variations, differences in experimental settings, the timing of administration, and species used.

### 2.2. PPARβ/δ

PPARβ/δ is the predominant PPAR subtype expressed in cardiac tissue [42]. Conditional cardiomyocyte-specific deletion of PPARβ/δ has been shown to induce myocardial lipid accumulation and cardiomyopathy, resulting in congestive heart failure with reduced survival. As the main mechanism for the cardioprotective action of PPARβ/δ, its leading role in maintaining normal fatty acid oxidation (FAO) was identified [43]. Animals with cardiomyocyte-specific deletion of PPARβ/δ were also examined in a study that aimed to establish an open-chest method for acquiring in vivo ^31^P nuclear magnetic resonance (NMR) cardiac spectra from mice at 4.7 Tesla. Interestingly, mice lacking PPARβ/δ in cardiomyocytes had even lower mean phosphocreatine (PCr)/adenosine triphosphate (ATP) ratios than control animals with myocardial infarction [44]. Given these important findings, it is astonishing that relatively few investigations focused on the implication of PPARβ/δ in myocardial infarction. Accordingly, nearly no clinical trials exist investigating the consequences of PPARβ/δ modulation in cardiovascular disease. The PPARβ/δ agonist GW501516 entered clinical trials to treat metabolic syndrome and diabetes at the beginning of 2000. These trials were stopped in 2007 due to multiple appearances of cancers in mice and rats [45], a finding which our group could confirm using either the PPARβ/δ agonist GW0742 or animals with conditional inducible vessel-specific overexpression of PPARβ/δ [46,47]. Currently, the angiotensin II receptor blocker telmisartan is one drug on the market that targets PPARβ/δ [48], as well as PPARγ [49,50]. Two clinical trials for telmisartan were completed: TRANSCEND (Telmisartan Randomized Assessment Study in ACE-Intolerant Subjects with Cardiovascular Disease) and ONTARGET (Ongoing Telmisartan Alone and in Combination with Ramipiril Global Endpoint Trial). No significant differences were observed between the groups in terms of primary and secondary outcomes, except for female patients who showed a 20% overall risk reduction for myocardial infarction [51]. It is, however, difficult to say if this beneficial effect of telmisartan can be attributed to angiotensin II receptor blockade or PPARβ/δ activation.

Concerning expression levels of PPARβ/δ after myocardial infarction, no changes could be observed after the infarction of rats [52]. The group around R. N. Willette examined the effects of the specific PPARβ/δ agonist GW610742X on the outcome after myocardial infarction in rats. The PPARβ/δ agonist did not ameliorate reduced left ventricular ejection fractions, and decreased phosphocreatine/adenosine triphosphate ratios, nor changed left ventricular weights or infarct sizes. In contrast, GW610742X normalized cardiac substrate metabolism after infarction and reduced right ventricular hypertrophy and pulmonary congestion [15]. Indirectly, cardioprotective functions of PPARβ/δ have been postulated in work from Li and coworkers. The authors investigated the beneficial effects of remote ischemic preconditioning (rIPC) for cardiac protection after myocardial infarction and the underlying molecular pathway. rIPC reduced infarct size and apoptosis and improved functional recovery. The authors demonstrated that protective effects of rIPC were mediated via the phosphoinositide 3-kinase (PI3K)/Akt/glycogen synthase kinase 3β (GSK3β) signaling pathway, which associates the nuclear accumulation of β-catenin and the up-regulation of its downstream targets E-cadherin and PPARβ/δ involved in cell survival [53]. Our group specifically aimed at elucidating a hypothetical benefit from vessel-specific overexpression of PPARβ/δ on recovery after myocardial infarction. Prompted by our earlier finding that PPARβ/δ agonist treatment induced a rapid increase in cardiac muscle mass and vascularization [54]. We also wanted to know if vascular specific PPARβ/δ overexpression would be sufficient to induce cardiac growth. In mice with inducible vascular specific overexpression of PPARβ/δ, we observed not only a rapid increase of cardiac vascularization but also a fast induction of cardiac growth, indicating that myocardial hypertrophy was due to enhanced angiogenesis. Vascular-specific PPARβ/δ overexpression impaired cardiac function, as evidenced by increased systolic and diastolic volumes, a reduced fractional shortening, and decreased ejection fractions. PPARβ/δ vessel-specific overexpression also increased capillary densities in the setting of myocardial infarction but failed to improve the outcome. We observed bigger infarct sizes, enhanced fibrosis, and significantly impaired echocardiographic parameters in the animals with the induction of vessel-specific overexpression of PPARβ/δ compared to controls. This indicates that the specific, unbalanced activation of PPARβ/δ only in the vasculature is not sufficient to protect against chronic ischemic heart disease [16,55]. Treatment with the PPARβ/δ agonist GW610742 after myocardial infarction in rats similarly has been reported to increase vessel densities and fibrosis, however, echocardiographic examinations revealed no differences between PPARβ/δ agonist treated animals and controls. GW610742 increased bone marrow-derived mesenchymal stem cell (MSC) recruitment in the heart and augmented the differentiation of fibroblasts into myofibroblasts. This was accompanied by increased serum platelet-derived growth factor B, stromal-derived factor-1 alpha, and MMP 9 levels. However, despite the enhanced angiogenesis, fibrosis, and myofibroblast differentiation in the early phase after infarction, the authors could not conclude the beneficial effects of PPARβ/δ activation on cardiac function after myocardial infarction [17]. In contrast to these studies, Magadum and coworkers observed a beneficial effect of PPARβ/δ activation on the outcome after myocardial infarction. It remains to be determined if different PPARβ/δ agonists used or different experimental settings might contribute to these discrepancies. Using an inducible mouse model with cardiomyocyte-specific overexpression of PPARβ/δ, Magadum and colleagues demonstrated smaller infarct sizes, enhanced cardiomyocyte proliferation, and improved functional parameters upon overexpression of PPARβ/δ in cardiomyocytes. They constated similar favorable effects by treating mice after ligation of the left anterior descending artery (LAD) with the PPARβ/δ agonist GW0742 [18]. These results partially confirm our hypothesis that a proper balance of PPARβ/δ activation in the different cardiac cell types may be important for potential cardioprotective effects of PPARβ/δ [16], and highlights the significance of cardiomyocyte PPARβ/δ expression for cardiac repair.

### 2.3. PPARγ

Thiazolinediones are the major class of PPARγ agonists, including rosiglitazone, pioglitazone, and troglitazone indicated for the treatment of type 2 diabetes. However, their usefulness has become controversial due to severe cardiovascular side effects [56,57]. In the PROactive (PROspective pioglitAzone Clinical Trial In macroVascular Events) study, enrolling patients with type 2 diabetes and pre-existing cardiovascular disease, pioglitazone increased the incidence of heart failure [58]. Especially, rosiglitazone has been associated with a higher risk for myocardial infarction and stroke [59]. However, the RECORD (rosiglitazone evaluated for cardiovascular outcomes in oral agent combination therapy for type 2 diabetes) trial could not confirm an increased risk for cardiovascular morbidity or mortality, but admitted inconclusive data about the incidence of myocardial infarction upon rosiglitazone therapy [60]. A science advisory from the American Heart Association and American College of Cardiology Foundation finally concluded that “thiazolidinediones should not be used with an expectation of benefit with respect to ischemic heart disease (IHD) events” [61]. Doney and colleagues investigated PPARγ variants in diabetic patients. They found an association between the *PPARG* Pro12Ala variant and decreased risk of myocardial infarction, while the C1431T genotype had the opposite effect. These polymorphisms might contribute to the conflicting results mentioned above; mechanistic consequences of the polymorphisms are currently unknown [62]. Later meta-analyses did not support a role of P12A polymorphism in the PPARγ gene in myocardial infarction or coronary heart disease risk [63]. Expression levels of PPARγ were found to be up-regulated after myocardial infarction in rats, however, increased PPARγ could not counteract the decrease in metabolic genes [52]. Experimental studies investigating a possible therapeutical potential for PPARγ agonists in ischemic heart disease started in 2001 with a report from Eliot H. Ohlstein’s group. Using ischemia/reperfusion manipulations in rosiglitazone treated rats, they observed reduced infarct sizes, an improvement of myocardial contractile dysfunction, less macrophage/neutrophil invasion, which correlated with decreased ICAM-1 and MCP-1 expression upon PPARγ activation with rosiglitazone. They ascribed the cardioprotective effect of rosiglitazone to the inhibition of inflammatory responses [19]. Similar results were obtained, as already mentioned in the PPARα section, by Wayman and colleagues, who in addition to rosiglitazone investigated the effects of ciglitazone, pioglitazone, 15D-PGJ_2_, and PGA_1_ [6], also by Ito and colleagues who focused only on pioglitazone [20]. The group of J. L. Mehta concentrated on the interplay of PPARγ and the renin-angiotensin system in myocardial ischemia. Rats treated with rosiglitazone or vehicle were subjected to ischemia (1 hr)/reperfusion (1 hr). Infarct sizes were smaller in the rosiglitazone group, and the authors found decreased ATR1 and increased ATR2 expression of angiotensin II (ANGII) receptors in the hearts from PPARγ agonist treated animals. This was accompanied by a down-regulation of mitogen-activated protein kinases (MAPKs) 42/44, indicating that the inhibition of MAPKs 42/44 by ATR2 ANGII represents one mechanism of rosiglitazone cardioprotective effects [21]. Similar beneficial results of rosiglitazone on left ventricular remodeling and cardiac function after myocardial infarction in rats were reported, however, in this study, no expression differences for ANGII, ATR1, and ATR2 were found [22]. In addition, in mice as well as in rabbits, cardioprotection by rosiglitazone after ischemia/reperfusion has been reported [24,25]. Comparable, administration of the PPARγ agonist pioglitazone after ischemia/reperfusion decreased myocardial necrosis, apoptosis, MMP2 levels, and improved systolic cardiac function in rats [23]. In rabbits treated with pioglitazone for seven days before ischemia/reperfusion, reduced infarct sizes, improved left ventricular function, and activation of (PI3K)/Akt and eNOS pathways were reported [26]. Zhang and colleagues also proposed activation of the (PI3K)/Akt pathway as a mechanism of rosiglitazone mediated cardioprotection in mice subjected to ischemia/reperfusion [27]. Curcumin [64], vitamin D [65], apigenin [66], the traditional Chinese medication qiliqiangxin [67], melatonin [68], the flavonoids chrysin [69,70] and fisetin [71], hesperitin derived from citrus fruits [72], and the purin alkaloide theacrine [73] have all been suggested to be cardioprotective in the setting of myocardial ischemia through the activation of PPARγ. Shinmura and colleagues used the PPARγ agonist pioglitazone to enhance the cardiomyogenic transdifferentiation potential of human marrow-derived mesenchymal stem cells (MSCs), which they injected two weeks after myocardial infarction in nude rats. Pioglitazone treated MSCs improved left ventricular function significantly more than non-treated MSCs [30]. Similarly, simultaneous pioglitazone treatment after MSC injection following myocardial infarction in rats ameliorated cardiac function more efficiently than MSC transplantation alone [31]. The group of Ferreira employed lysophosphatidic acid (LPA) to enhance the survival of human umbilical cord blood-derived hematopoietic stem/progenitor cells to boost the regenerative potential in the setting of myocardial infarction. LPA enhanced survival through activation of PPARγ and pro-survival extracellular signal related kinases (ERK) and Akt signaling pathways and inhibition of mitochondrial apoptotic pathway. Injection of LPA treated cells improved cardiac fractional shortening and ejection fraction parameters after myocardial infarction [32]. The importance of PPARγ expression in myeloid cells for cardiac repair after infarction has been supported by the group of Duan, which analyzed the outcomes of myocardial infarctions in mice with myeloid specific knockout for PPARγ. Pioglitazone increased the repair potential of adipose tissue-derived regenerative cells (ADRCs) upon grafting on the anterior left ventricular wall two weeks after myocardial infarction in rats as reflected by improved functional cardiac parameters [36]. Myeloid PPARγ knockout animals had bigger infarct sizes, worse cardiac functional parameters, and enhanced oxidative stress and immune responses compared to their control counterparts [34]. The angiotensin II receptor blocker telmisartan, which also targets PPARγ [50], has been evaluated in an experimental model of isoproterenol (a synthetic non-selective β-adrenoceptor agonist) induced myocardial injury. Telmisartan lowered left ventricular end-diastolic pressure and improved biochemical, histopathological, and ultrastructural parameters [74]. The same group reported similar beneficial effects of telmisartan in diabetic rats with isoproterenol induced myocardial injury, which could be counteracted using the PPARγ antagonist GW9662 [75]. In a profound study using LAD ligation in rats, Maejima and colleagues demonstrated that Telmisartan attenuated unfavorable left ventricular remodeling after myocardial infarction, but did not influence infarct sizes or blood pressure, indicating that the favorable effects were blood pressure independent. Furthermore, co-administration of GW9662 abolished the beneficial effects of telmisartan on left ventricular remodeling, further suggesting PPARγ agonistic activity of this drug [29]. In 2010, Tao and colleagues aimed at solving the discrepancies of experimental and clinical studies regarding the effects of PPARγ agonists of the thiazolidinedione class in cardioprotection. They used adiponectin (an adipocytokine secreted from adipose tissue) knockout and wildtype mice to show that anti-oxidative, anti-ischemic, anti-apoptotic, and cardioprotective actions of the PPARγ agonist rosiglitazone depend on normal adiponectin (APN) levels. Rosiglitazone improved post-MI survival rate and cardiac function in wildtype mice after ligation of the LAD, but not in APN knockout animals. The PPARγ agonist further reduced infarct sizes, apoptosis, and oxidative stress in normal mice, however, failed to produce these beneficial effects in the APN knockout group and provoked an enhanced superoxide production only in the APN deficient hearts. Treatment with a superoxide dismutase mimic reversed the detrimental effects of rosiglitazone in APN knockout animals, indicating that the anti-oxidant effect of rosiglitazone relies on APN. Adiponectin is down-regulated in obesity related diseases such as Diabetes type 2 or coronary artery disease, which might partially explain the unfavorable outcomes in clinical studies using rosiglitazone in such pathologies [28]. An original approach demonstrated that nanoparticle (NP) mediated targeting of pioglitazone to monocytes/macrophages, but not systemic intravenous treatment with pioglitazone solution, ameliorated ischemia/reperfusion injury, and cardiac remodeling. Pioglitazone-NPs antagonized monocyte/macrophage-mediated acute inflammation and promoted cardiac healing after myocardial infarction as also evidenced by improved cardiac functional parameters [35]. In addition, microRNA studies focused on PPARγ: Zhao and colleagues demonstrated that PPARγ promotes microRNA (miR) 711 expression after myocardial infarction in rats, which in turn induced downregulation of the chaperone calnexin leading to enhanced cardiac apoptosis due to endoplasmatic reticulum stress [76]. Downregulation of miR-130 expression has been shown to promote PPARγ-mediated cardioprotective effects by suppressing inflammation and myocardial fibrosis [77].

Although, as already mentioned in the PPARα chapter, several clinical trials testing dual PPARα/γ agonists had either to be stopped due to increased rates of heart failure as the AleCardio trial for Aleglitazar [78] or due to elevation of serum creatinine, bodyweight increase, and edema formation with tesaglitazar [79], or major adverse cardiovascular events as for muraglitazar [37], experimental research continued on the concept of dual PPARα/γ agonism. In rats with myocardial infarction, the dual PPARα/γ agonist TZD18 improved left ventricular function and increased the expression of enzymes related to myocardial energy metabolism and the content of high energy phosphate in mitochondria [33].

In conclusion, although PPARγ agonists offer benefits in the treatment of diabetes and atherosclerosis, known risk factors associated with cardiovascular disease, they also have deleterious effects such as increased risk incidence of myocardial infarction and heart failure. Their clinical use remains, therefore, limited.

## 3. Conclusions

PPAR agonists have very different and sometimes conflicting clinical benefit and adverse event profiles. The rationale for metabolic therapy to remedy cardiovascular disease and dysfunction is clearly understandable given the importance of PPARs in the control of lipid and energy metabolism. However, taking into account the serious side effects of PPAR activators, such as the higher incidence of myocardial infarction observed in clinical trials, further work is required to delineate the intricacies of modulating their activity for optimal therapeutic benefit.

## Figures and Tables

**Table 1:** Type of peroxisome proliferator-activated receptors (PPAR) modulation and experimental settings used in animal studies.

Concerned PPAR	Type of PPAR Modulation	Experimental Setting	Species	Outcome for Myocardial Infarction	Citation
PPARα	Agonists, Clofibrate, WY14643, 30 min before, ischemia	ischemia 25 min, reperfusion 2 h	rats	reduced infarct sizes	[6]
PPARα	agonist GW7647, 2 days and 1 h before ischemia	ischemia 30 min reperfusion 24 h	mice	reduced infarct sizes, improvement of myocardial contractile dysfunction	[7]
PPARα	cardiac specific overexpression, knockout	ischemia 18 min reperfusion 40 min	mice	worsened cardiac function, improved cardiac function	[8]
PPARα	agonist fenofibrate, 12 weeks after MI	myocardial infarction	rats	unchanged leftventricular dysfunction	[9]
PPARα	agonist fenofibrate, 4 weeks before ischemia	ischemia 90 min reperfusion 120 min	pigs	unchanged infarct sizes and cardiac function	[10]
PPARα	agonist WY14643, 35 min before ischemia	ischemia 35 min reperfusion 2 h	Goto-Kakizaki, rats	reduced infarct sizes	[11]
PPARα	cardiomyocyte specific overexpression	repetitive, brief I/R during several days	mice	impaired ventricular function	[12]
PPARα	agonist clofibrate, 7 days after MI for 7 days	myocardial infarction	rats	reduced left ventricular dilatation, LVEF unchanged	[13]
PPARα	agonist fenofibrate, ginsenoside Rb3, 7 days after MI	myocardial infarction	mice	Ejection fractions and fractional shortening increased	[14]
PPARβ/δ	agonist GW610742X, 6–9 weeks after MI	myocardial infarction	rats	infarct sizes and EF fractions unchanged	[15]
PPARβ/δ	conditional inducible vessel specific overexpression, started 1 week before MI	myocardial infarction	mice	bigger infarct sizes, worse functional parameters	[16]
PPARβ/δ	agonist GW610742X, after MI every 3 days	myocardial infarction	rats	unchanged functional parameters	[17]
PPARβ/δ	conditional inducible cardiomyocyte specific overexpression started 1 week before MI, agonist GW0742 after MI for 2 weeks	myocardial infarction	mice	smaller infarct sizes, better functional parameters	[18]
PPARγ	agonist rosiglitazone, before occlusion and after reperfusion	ischemia 30 min, reperfusion 24 h	rats	smaller infarct sizes, better functional parameters	[19]
PPARγ	agonists rosiglitazone, ciglitazone, pioglitazone, 15D-PGJ_2,_ PGA_1,_ 30 min before, ischemia	ischemia 25 min, reperfusion 2 h	rats	substantial reduction of infarct sizes, most pronounced with 15D-PGJ_2_	[6]
PPARγ	agonist pioglitazone 7 days before ischemia	ischemia 30 min, reperfusion 24 h	rats	smaller infarct sizes	[20]
PPARγ	agonist rosiglitazone, fed regularly, before occlusion	ischemia 1 h, reperfusion 1 h	rats	smaller infarct sizes	[21]
PPARγ	agonist rosiglitazone after MI for 8 weeks	myocardial infarction	rats	better functional parameters	[22]
PPARγ	agonist pioglitazone after occlusion	ischemia 30 min, reperfusion 30 min or 120 min	rats	better systolic function, less necrosis	[23]
PPARγ	agonist rosiglitazone, during reperfusion	ischemia 20 min, reperfusion 28 days	mice	smaller infarct sizes	[24]
PPARγ	agonist rosiglitazone, before occlusion	ischemia 1 h, reperfusion 4 h	rabbits	smaller infarct sizes	[25]
PPARγ	agonist pioglitazone 7 days before occlusion	ischemia 30 min, reperfusion 48 h	rabbits	smaller infarct sizes improved left ventricular function	[26]
PPARγ	agonist rosiglitazone, for 14 days, before occlusion	ischemia 30 min, reperfusion 2 h	mice	smaller infarct sizes, ventricular fibrillation reduced	[27]
PPARγ	agonist rosiglitazone, for 3 days, before MI	myocardial infarction	wildtype and APN knockout mice	smaller infarct sizes, better survival and functional parameters in wildtype, but not in APN ko mice	[28]
PPARγ	Telmisartan, ortelmisartan+ antagonist GW9662, for 28 days after MI	myocardial infarction	rats	mortality reduced, left ventricular systolic function improved, GW9662 abolished these effects	[29]
PPARγ	agonist pioglitazone treated MSCs, 2 weeks after MI	myocardial infarction	nude rats	improvement of left ventricular function	[30]
PPARγ	agonist pioglitazone, 2 weeks after MI and MSC injection	myocardial infarction	rats	better functional parameters	[31]
PPARγ	injection of LPA treated progenitor cells after MI	myocardial infarction	rats	better functional parameters	[32]
PPARα/γ	dual agonist TZD18 after MI	myocardial infarction	rats	improvement of left ventricular function	[33]
PPARγ	myeloid specific knockout	myocardial infarction	mice	increased infarct sizes, worse functional parameters	[34]
PPARγ	agonist pioglitazone	ischemia 30 min in mice, 60 min in pigsreperfusion different time points	mice, pigs	decreased infarct sizes, better functional parameters	[35]
PPARγ	pioglitazone treated adipose tissue-derived regenerative cells	myocardial infarction	rats	improved functional parameters	[36]

## Abbreviations

APN	adiponectin
ADRCs	adipose tissue-derived regenerative cells
ATP	adenosine triphosphate
Akt	serine/threonine-specific protein kinase
CPT 1	carnitine palmitoyltransferase 1
EF	ejection fraction
ERK	extracellular signal related kinases
FAO	fatty acid oxidation
FS	fractional shortening
eNOS	endothelial NO synthetase
GRb-3	ginsenoide Rb-3
GSK3β	glycogen synthase kinase 3β
HO-1	hemeoxygenase-1
IL	Interleukin
ICAM	intercellular adhesion molecule
iNOS	inducible nitric oxide synthase
LAD	left anterior descending artery
LPA	lysophosphatidic acid
LVEF	left ventricular ejection fractions
MMP	matrix metallopeptidase
MAPK	Mitogen-activated protein kinase
MCP-1	monocyte chemoattractant protein-1
MI	Myocardial infarction
miR	micro RNA
MSCs	marrow-derived mesenchymal stem cells
NP	nanoparticle
NF-κB	nuclear factor kappa-light-chain-enhancer of activated B cells
NMR	nuclear magnetic resonance
NO	Nitric oxide
PCr	phosphocreatine
PI3K	phosphoinositide 3-kinase
rIPC	remote ischaemic preconditioning
SNPs	single-nucleotide polymorphisms
VCAM	vascular cell adhesion molecule
